# Addressing the burden of cervical cancer for Indigenous women in Latin America and the Caribbean: a call for action

**DOI:** 10.3389/fpubh.2024.1376748

**Published:** 2024-05-14

**Authors:** Claire Muslin

**Affiliations:** One Health Research Group, Faculty of Health Sciences, Universidad de las Américas, Quito, Ecuador

**Keywords:** cervical cancer, HPV, indigenous, Latin America, Caribbean

## Abstract

Cervical cancer, primarily caused by human papillomavirus (HPV) infection, poses a significant global health challenge. Due to higher levels of poverty and health inequities, Indigenous women worldwide are more vulnerable to cervical cancer than their non-Indigenous counterparts. However, despite constituting nearly 10% of the population in Latin America and the Caribbean (LAC), the true extent of the burden of cervical cancer among Indigenous people in this region remains largely unknown. This article reviews the available information on cervical cancer incidence and mortality, as well as HPV infection prevalence, among Indigenous women in LAC. The limited existing data suggest that Indigenous women in this region face a heightened risk of cervical cancer incidence and mortality compared to non-Indigenous women. Nevertheless, a substantial knowledge gap persists that must be addressed to comprehensively assess the burden of cervical cancer among Indigenous populations, especially through enhancing cancer surveillance across LAC countries. Numerous structural, social and cultural barriers hindering Indigenous women’s access to HPV vaccination and cervical cancer screening worldwide have been identified and are reviewed in this article. The discussion highlights the critical role of culturally sensitive education, community engagement, and empowerment strategies in overcoming those barriers. Drawing insights from the success of targeted strategies in certain high-income countries, the present article advocates for research, policies and healthcare interventions tailored to the unique context of LAC countries.

## 1 Introduction

Cervical cancer is caused by human papillomavirus (HPV) infection, the most common sexually transmitted viral infection worldwide (1, 2). It has been estimated that, in the United States, more than 80% of sexually active women and men will acquire at least one HPV infection by the age of 45 years (3). In women, 90% of incident genital HPV infections clear within 2 years without any clinical impact (4). However, when persistence occurs, HPV becomes a risk factor for malignant transformation. Out of the more than 200 HPV types identified, 12 high-risk HPVs are responsible for virtually all cases of cervical cancers (5, 6).

Cervical cancer is one of the few cancers preventable through both vaccination and screening. Several prophylactic HPV vaccines are available in most countries and the World Health Organization (WHO) currently recommends one or two-dose vaccination of girls aged 9 to 14 years (7). As various high-risk HPV types are not covered by the vaccines, high-quality screening programs are also paramount to prevent cervical cancer. Screening can detect premalignant and early malignant lesions that can be treated with a very good prognosis (8). Three different methods can be used to identify women who have, or are at risk of, cervical precancerous lesions and early invasive cancer: (1) detection of genital high-risk HPV infection through a DNA-based test on cervical or vaginal samples; (2) microscopic examination of cervical exfoliated cells, known as the Papanicolaou (Pap) cytology test; or (3) visual inspection of the cervix with acetic acid, in low-resource settings (7). In high-income countries, the implementation of screening and treatment programs since the 1950s followed by the introduction of HPV vaccination since 2006 has led to a dramatic reduction in both the prevalence of HPV vaccine-types and closely related HPV types and the incidence and mortality of cervical cancer (9–14). Before the effectiveness of these prevention measures, the WHO launched in 2020 a strategy for the global elimination of cervical cancer, with three main objectives to be reached by 2030: 90% vaccination rates, 70% screening rates and treatment for 90% of the invasive cancers for women in all countries (15).

Unfortunately, challenges persist in low- and middle-income countries in implementing cervical cancer screening programs and ensuring access to HPV vaccines. As a result, these countries currently bear the largest burden of this preventable disease, with cervical cancer incidence and mortality worldwide strongly correlated with country income level, human development index and living standards (16, 17). At present, cervical cancer ranks fourth in cancer incidence and mortality among women globally (18). In 2020 alone, an estimated 604,000 new cases and 342,000 deaths were reported, with over 80% of these cases occurring in low- and-middle-income countries (18, 19). In Latin America and the Caribbean (LAC), most of the countries have implemented cervical screening and HPV vaccination programs, and trends in cervical cancer incidence and mortality have decreased over the past 20 years (20, 21). However, several barriers to screening access and vaccination uptake persist (22, 23), and the age-standardized incidence and mortality rates in LAC in 2018 were 14.6 and 7.1 per 100,000, respectively, ranking second after the African region and slightly higher than the global rates (13.1 and 6.9, respectively) (20). In 2020, cervical cancer remained the leading cause of cancer death in women in Belize, Honduras, Nicaragua, Bolivia and Paraguay (18).

An estimated 58 million Indigenous people lived in Latin America in 2018, accounting for 10% of the total population of the subregion (24). While no formal definition has been adopted in international law, a contemporary and inclusive understanding of “Indigenous peoples” has been developed and includes those who self-identify and are identified as indigenous, exhibit historical continuity with precolonial societies, possess distinct social, economic, or political systems, maintain unique languages, cultures, and beliefs, and constitute nondominant groups of society (25, 26). In most LAC countries, the determination of indigenous status in population censuses relies on self-identification by individuals (26). The distribution of Indigenous people varies widely across LAC, both in terms of absolute numbers and relative proportions (Table 1). Guatemala, Bolivia and Peru have the highest proportions of indigenous population, with 43.6, 41.5 and 26.0%, respectively. Meanwhile, Mexico is the country with the largest number of indigenous individuals, totaling around 25 million.

**Table 1:** Indigenous population, recording of indigenous status by cancer registries, and prevalence of cervical intraepithelial lesions and HPV infection among Indigenous women in Latin America and the Caribbean.

Country	Indigenous population^a^ (% of general population)		Cancer registries recording indigenous status^d^	Prevalence of cervical intraepithelial lesions among Indigenous women, sample size n, study population, (study reference)^e^		Prevalence of cervical HPV infection among Indigenous women, sample size n, study population, (study reference)^f^	
**South America**							
Argentina	1,306,730	(2.9%)	No	4.3% (0.5% CC), n = 207, Guarani 7.9% (0.4% CC), n = 227, Pilaga	(27) (28)	64.3%, n = 207 Guarani 46.7%, n = 227 Pilaga	(27) (28)
Bolivia	4,910,670	(41.5%)	No	ND		ND	
Brazil	1,227,642	(0.6%)	Yes	4.0% (0.9% CC), n = 423 15.3%, n = 72, Parakana 10.7%, n = 84, Panará 5.1% (0.4% CC), n = 275 Yanomami 1.8%, n = 332, Macuxi, Wapishana 8.4%, n = 395, Quilombo 10.0%, n = 241 1.8%*, n = 3,231	(29) (30) (31) (32) (32) (33) (34) (35)	14.3%, n = 42, Parakana 42.9%, n = 49, Parakana 45.9%, n = 305, Yanomami 34.5%, n = 359, Macuxi, Wapishana 12.6%, n = 395, Quilombo	(30) (36) (32) (32) (33)
Chile	2,382,333	(12.4%)	No	ND		ND	
Colombia	2,255,697	(4.4%)	No	ND		63.8%*, n = 47 31.1%, n = 280	(37) (38)
Ecuador	1,301,887	(7.7%)	No	2.0%, n = 100, Kichwa	(39)	34.0%, n = 100, Kichwa 28.3%, n = 396, Kichwa, Shuar	(39) (40)
French Guiana	10000^b^	(3.5%)	No	ND		ND	
Guyana	78,492	(10.5%)	Yes	11.2% (0.8% CC), n = 2,250	(41)	19.3%, n = 1,423	(41)
Paraguay	140,206	(2.3%)	No	13.2%, n = 129, Ache, Ava Guaraní, Mbya Guaraní	(42)	23.2%, n = 181	(43)
Peru	8,673,449	(26.0%)	No	13.4%, n = 307, Shipibo-Konibo 2.1% ǂ, n = 48, Bora	(44) (45)	31.6%, n = 307 Shipibo-Konibo 35.4%(ns), n = 48, Bora	(44) (45)
Suriname	20,344	(3.8%)	No	ND		ND	
Uruguay	83,644	(2.4%)	Yes	ND		ND	
Venezuela	775,034	(2.7%)	No	1.8%, n = 57, Yekwana, Piaroa, Arawaco 14.3%(ns), n = 35, Eñepa 11.0%(ns), n = 82, Piaro	(46) (47) (48)	35.1%, n = 57 Yekwana, Piaroa, Arawaco 45.7%*, n = 35, Eñepa 72.0%(ns), n = 82, Piaro	(46) (47) (48)
**North and Central America**							
Belize	56,146	(17.4%)	No	ND		ND	
Costa Rica	123,337	(2.4%)	No	ND		ND	
El Salvador	13,037	(0.2%)	No	ND		ND	
Guatemala	7,956,939	(43.6%)	Yes	8.1% (0.5% CC), n = 222	(49)	ND	
Honduras	784,913	(7.8%)	No	ND		ND	
Mexico	25,280,302	(19.4%)	Yes	70.4%, n = 108, Nahuatl	(50)	70.4%, n = 108, Nahuatl	(50)
Nicaragua	422,250	(6.3%)	No	ND		ND	
Panama	698,114	(17.2%)	No	ND		ND	
**Caribbean** ^**c**^							
British Virgin Islands	177	(0.6%)	Yes	ND		ND	
Dominica	2,576	(3.7%)	No	ND		ND	
S^t^ Vincent & the Grenadines	3.280	(3%)	No	ND		ND	

^a^Sources: Economic Commission for Latin America and the Caribbean (ECLAC), The sociodemographic impacts of the COVID-19 pandemic in Latin America and the Caribbean (LC/CRPD.4/3), Santiago, 2022. Argentina data: Instituto Nacional de Estadística y Censos, Censo Nacional de Población, Hogares y Viviendas 2022. Belize data: The Statistical Institute of Belize, Belize Population and Housing Census 2010. Brazil data: Instituto Brasileiro de Geografia e Estatística, Censo 2022. British Virgin Islands data: Government of the Virgin Islands, 2010 Population and Housing Census Report. Dominica data: Central Statistics Office of Dominica, 2011 Population and Housing Census. Ecuador data: Instituto Nacional de Estadística y Censos, Censo Ecuador 2022. Guyana data: Bureau of Statistics, 2012 Census. Panama data: Instituto Nacional de Estadística y Censo, XII Censo Nacional de Población y VIII de Vivienda 2023. Paraguay data: Instituto Nacional de Estadística, Resultados Preliminares del IV Censo Nacional de Población y Viviendas para Pueblos Indígenas 2022. Saint Vincent & the Grenadines data: Statistical Office, Government of Saint Vincent and the Grenadines, Population and Housing Census Report 2012. Suriname data: Algemeen Bureau voor de Statistiek, 2012 Census. ^b^Due to the prohibition of ethnic statistics by the French Constitution, only estimates from unofficial studies collected by the Associations for the Defense of Indigenous Peoples of French Guiana are available. ^c^Caribbean countries with distinct indigenous communities still in existence. ^d^Sources: (26, 51–56). ^e^Cervical intraepithelial lesions include: Atypical squamous cell of unknown significance (ASCUS), Low grade squamous cell intraepithelial lesion (LSIL); High grade squamous cell intraepithelial lesion (HSIL) and invasive cervical cancer (CC). Ethnics of the study population specified when available. ^f^Prevalence of any-type HPV. ^*^Rate significantly higher than in the non-Indigenous comparison group. (ns) Rate not significantly higher than in the non-Indigenous comparison group. ǂRate significantly lower than in the non-Indigenous comparison group. ND: No data.

Despite the inherent cultural, geographic, and genetic diversity among the more than 800 distinct ethnic groups in LAC (57), Indigenous peoples share a common history of colonization and ongoing dispossession of traditional lands, resources and practices (58). Consequently, Indigenous peoples suffer the greatest structural inequalities in Latin America and are over-represented among the socially disadvantaged in almost every country (59). According to estimations from 2020, 45.5% of Indigenous people in Latin America live in poverty and 7.1% in extremely poverty, more than twice the rates for non-Indigenous people in the region (60). Furthermore, in most LAC countries, indigenous people continue to primarily live in rural areas associated with their ancestral territories, and when they live in cities, it is often in extreme poverty in marginal areas, with trouble accessing basic services and decent jobs (59). One consequence of this marginalization is that Indigenous people in LAC have disproportionally worse health than their nonindigenous counterparts (61). Specifically, Indigenous women are directly impacted by the strong association between poverty and cervical cancer.

The primary objective of this perspective article is to examine the existing data on the burden of cervical cancer among Indigenous women in LAC. Moreover, it aims to identify the barriers potentially hindering their access to HPV vaccination, cervical cancer screening and treatment services. Finally, the article explores proven strategies from high-income countries that have successfully addressed similar challenges, with a view toward their potential applicability within LAC nations.

The articles included in this paper were obtained through a systematic literature search conducted on the PubMed Medline database from its inception to February 2024, using the following terms: (“cervical cancer” OR “papillomavirus”) AND “indigenous.” All abstracts retrieved were reviewed and assessed to identify relevant studies, including peer-reviewed original articles, meta-analyses, reviews, and reports published in English, Spanish or Portuguese. Additional articles were gathered from the references of selected publications. Studies were excluded if they: (1) were unrelated to human papillomavirus infection or cervical cancer; (2) did not clearly define Indigenous people as the study population; (3) were not published in peer-reviewed journals. Furthermore, demographic and epidemiological data specific to each LAC country, including cancer registries and population censuses, were manually searched for on official governmental websites. As a result, findings were categorized and presented through a narrative synthesis in subsequent sections.

## 2 The burden of cervical cancer among Indigenous women in Latin America and the Caribbean

Because they frequently experience both ethnic and gender discrimination, Indigenous women often face the most profound structural inequalities, especially concerning poverty, education, and healthcare (59). Consequently, they are particularly vulnerable to cervical cancer. Over the last decades, numerous studies have extensively reported and characterized the disproportionately high burden of cervical cancer incidence and mortality among Indigenous women compared to non-Indigenous ones in high-income countries, namely Australia, New Zealand, Canada and the United States (62–68). A higher prevalence of HPV infection has also been observed among Indigenous women compared to the general population in these countries (69–72).

On the contrary, in the LAC region, the actual magnitude of the burden of cervical cancer among Indigenous women remains largely unknown and may be substantially under-estimated due to insufficient data collection. To the best of available information, indigenous status is currently recorded in the population-based cancer registries of only six out of the 24 LAC countries with Indigenous population (Table 1) (26, 51–56). However, none of these six countries publicly provide cancer rates for Indigenous people in their registry reports. Two recent studies have estimated cervical cancer mortality among Indigenous women in Brazil, reporting a mean age-standardized mortality rate of 6.7 per 100,000 between 2000 and 2020, the highest among all ethnic groups in the country and corresponding to a significant 80% increase in cervical cancer death risk compared to white women (73, 74). Regarding cervical cancer incidence, a study conducted on the Indigenous population in the State of Acre, located in the Brazilian Western Amazon, reported that, between 2000 and 2012, cervical cancer was the most frequent neoplasm among indigenous women, and cervical cancer incidence was significantly higher compared to the reference population (Standardized Incidence Ratio: 4.49) (75). Similarly, an analysis of the population-based cancer registry of Guyana from 2000 to 2009 highlighted that cervical cancer was significantly more common among Indigenous Amerindian women compared to other ethnic groups (53). Based on the available information, the assessment of cervical cancer risk among Indigenous women in the rest of LAC relies exclusively on studies investigating the prevalence of cervical HPV infection and cervical intraepithelial lesions within Indigenous populations. Table 1 summarizes the findings of these studies. Reported rates of any-type HPV infection among Indigenous women in LAC are notably high, ranging from 12.6 to 72.0%. Importantly, the majority of studies (15/19) identified higher cervical HPV prevalence rates compared to the meta-analysis estimates for the general populations of Brazil and Latin America, respectively 25.41 and 16.1% (76, 77). Furthermore, a wide range of cervical intraepithelial lesions prevalence rates have been reported among Indigenous women across LAC, varying from 1.8 to 15.3%, except for a study in Mexico which documented an exceptionally high prevalence of 70.4% among Nahuatl women (50). Notably, nine out of the 20 identified studies reported a prevalence of cervical lesions exceeding 10%, while the prevalence of cervical lesions in women worldwide generally remains under 10% (78).

In conclusion, the currently available data suggest that Indigenous women in LAC face a heightened risk of cervical cancer incidence and mortality, mirroring trends observed in Indigenous populations in high-income countries and reflecting the health inequities they experience. However, there remains a significant lack of data on cervical cancer trends among Indigenous women in most LAC countries, including countries like Guatemala, Bolivia, and Mexico, which have some of the highest proportions of Indigenous populations in the region. The wide variations observed in the prevalence of HPV infection and cervical abnormalities may reflect disparities in cervical cancer risk linked to the heterogeneity of Indigenous groups across LAC, including differences in geographical location, degree of isolation, and social and sexual behaviors (35). Consequently, further research is imperative to comprehensively assess the burden of cervical cancer among Indigenous women both regionally and within each LAC country.

## 3 Barriers to cervical cancer screening and HPV vaccination among Indigenous women

Indigenous women worldwide exhibit a higher prevalence of HPV infection as well as increased cervical cancer incidence and mortality rates compared to the general population. This disproportionate burden of cervical cancer in Indigenous women in both high- and low-and-middle-income countries could be explained by substantially lower rates of HPV vaccination (79–81), lower cervical screening coverage and longer time to clinical investigation (67, 82–85) in Indigenous women compared to non-Indigenous women. Numerous multifaceted barriers have been identified that hinder proper access for Indigenous women to HPV vaccination and cervical cancer screening and treatment (Table 2). While many of these obstacles are not exclusive to Indigenous individuals, the intricate interplay of structural, social and cultural barriers in cervical cancer prevention, diagnosis and treatment uniquely challenges Indigenous women, contributing to their disproportionate burden of this disease.

**Table 2:** Main barriers to HPV vaccination and cervical cancer screening in Indigenous population globally.

Country – Study population		Structural barriers			Social barriers		Cultural barriers					Ref.
	Participant characteristics, sample size	Remoteness	Individual cost	Resource constraints in healthcare systems^a^	Limited knowledge about HPV, HPV vaccine and/or cervical cancer	Limited health literacy^b^	Sex and cultural taboos^c^	Mistrust in healthcare systems and/or in vaccine^d^	Language	Fear of pain	Culturally inappropriate health education delivery	
**Barriers to HPV vaccination**												
USA - American Indians (AI)												
	M & F, parents/caregivers, 260					X		X				(86)
	F, parents/caregivers, 50				X	X		X				(87)
	M & F, healthcare providers, 35	X			X		X	X				(88)
	M & F, healthcare providers, 31			X	X			X				(89)
	M & F, healthcare providers, vaccine eligible individuals (all ages), parents/caregivers, 73			X			X	X				(90)
	M & F, vaccine eligible individuals (>18 years), 57				X	X						(91)
	F, community members, 194				X							(92)
USA - Alaska Natives (AN)												
	F, vaccine eligible individuals (<18 years), 79				X			X		X		(93)
	M & F, parents/caregivers, 79				X			X				(94)
USA - AI/AN												
	M & F, healthcare providers, 319				X		X	X				(95)
USA - Native Hawaiians												
	M & F, parents/caregivers, 189					X		X				(96)
Canada - First Nations												
	M & F, community members, 24		X				X	X				(97)
Papua New Guinea - Papuans												
	M & F, community members, 208					X	X	X			X	(98)
Peru - Quechuas												
	F, parents/caregivers, 192						X	X	X			(99)
**Subtotal**		**1**	**1**	**2**	**8**	**5**	**6**	**12**	**1**	**1**	**1**	**14**
**Barriers to cervical cancer screening**												
Canada - Inuit												
	F, community members, 27					X						(100)
	F, community members, 175				X		X			X		(101)
Canada - First Nations												
	F, community members, 69 M & F, healthcare providers, 16						X	X				(102)
	M & F, healthcare providers, 18	X		X		X						(103)
	F, community members, 8						X	X				(104)
Australia - Aboriginal and Torres Strait Islander peoples												
	F, community members, 29						X	X				(105)
	M & F, healthcare providers, 13			X								(106)
	M & F, community members, 368, healthcare providers, 179		X	X	X			X				(107)
New Zealand - Māori												
	F, community members, 397						X			X		(108)
Papua New Guinea - Papuans												
	M & F, community members, 208				X		X		X		X	(109)
Mexico - Mam, Nahua and Huichol												
	F, community members, 122	X		X	X				X		X	(110)
Dominica - Kalinago												
	M & F, community members, 42				X							(111)
Guatemala - Maya, Garifuna, Xinca												
	F, community members, 5,728	X	X				X		X		X	(82)
Ecuador - Kichwa												
	F, community members, 28							X	X			(112)
Peru - Shipibo, Quechua												
	F, community members, 18	X	X									(113)
**Subtotal**		**4**	**3**	**4**	**5**	**2**	**7**	**5**	**4**	**2**	**3**	**15**
**Total Barriers**		**5**	**4**	**6**	**13**	**7**	**13**	**17**	**5**	**3**	**4**	**29**

F: female; M: male. Details on identified barriers: ^a^Lengthy waiting time to get an appointment, Shortages of supplies, Shortages of female healthcare providers, Lack of funding, Competing clinical priorities, Workforce capacity limitations, Lack of recall and follow-up systems. ^b^Participants reported that the healthcare provider did not mention the vaccine, Participants reported not knowing enough about the vaccine to be able to make a decision, not knowing where to seek information. ^c^Concerns that HPV vaccine may encourage earlier or riskier sexual activity, Negative perceptions regarding sexual health promotion, Discomfort and vulnerability, Triggering distress and trauma, Stigma associated with sexually transmitted diseases, Lack of privacy and control, Women requiring permission from partner to go to the clinic, Fear of cancer. ^d^Concerns about vaccine long-term safety and side-effects, Suspicion of Western medicine and preference for the use of traditional medicine, Complicated relationships with healthcare providers.

### 3.1 Structural barriers

Structural impediments play a pivotal role in limiting cervical cancer screening for Indigenous women. Most of them live in rural areas, where distance from health care centers and practitioners, coupled with the lack of personal resources, limit access to appropriate and timely health care, and pose challenges to screening, diagnosis and treatment (82, 88, 97, 103, 107, 110, 113). Furthermore, fragmentation of healthcare systems and economic constraints present additional challenges to the implementation of specific programs for prevention and control of cervical cancer among Indigenous women (23, 89, 90, 103, 106, 107, 110) (Table 2).

### 3.2 Social determinants

A complex interplay of social determinants, including limited health literacy, low education rates, low socioeconomic status, and isolation, exacerbates barriers to health services. Lack of awareness of cervical cancer and limited understanding of HPV role in its etiology has been identified as one of the main barrier contributing to low screening rates and HPV vaccination coverage among Indigenous women worldwide (see Table 2).

### 3.3 Cultural factors

Indigenous women often face discrimination and challenges in accessing culturally safe cervical screening and treatment services. Cultural differences and language barriers contribute to communication challenges with health care providers (82, 98, 99, 109, 110, 112). Furthermore, colonization disrupted Indigenous knowledge systems, de-valuing traditional practices and creating historical mistrust impacting their willingness to undergoing gynecological inspections and cervical cytology invasive procedure, as well as to participate in vaccination campaign (81, 114). Thus, mistrust in healthcare systems and HPV vaccines appears to be the first barrier to cervical cancer prevention and control among Indigenous women globally (see Table 2). Finally, community sensitivities regarding sexual health promotion and sexually transmitted diseases were also identified as one of the main limitation to both HPV vaccination and cervical cancer screening.

## 4 Strategies to improve cervical screening and HPV vaccination uptake for Indigenous women in Latin America and the Caribbean: recommendations for action

Cervical cancer poses a significant public health challenge for Indigenous women globally, demanding targeted strategies in response to the unique cultural and systemic factors hindering cervical screening and HPV vaccination uptake in Indigenous populations. Canada, Australia and New Zealand have implemented specific programs and initiatives of cancer screening and/or HPV vaccination that are showing promising results in reducing HPV-related diseases among Indigenous populations (97, 115–121). While taking example from these successful policies and strategies, it is imperative to devise solutions adapted to the socio-economic realities of LAC countries and the cultural diversity within Indigenous populations residing in the region.

### 4.1 Cultural sensitivity and education

Works in collaboration within Métis Nation communities in Canada have highlighted the importance of culturally-sensitive educational approaches to promote cervical cancer awareness and vaccination and to address misconceptions (115). Implementing culturally appropriate programs and ensuring that health care practitioners receive adequate support and resources to be able to incorporate cultural components into the delivery of information have been demonstrated to improve screening rates and HPV vaccination uptake among Indigenous women and adolescents in high-income countries (100, 102, 106, 114, 122, 123).

### 4.2 Community engagement and empowerment

Recognizing the influence of cultural factors on decision-making is crucial. Research from Canada and Australia has demonstrated that indigenous leadership plays a pivotal role in shaping research priorities, providing policy guidance, and developing strategies that are acceptable, appropriate and sustainable for Indigenous communities (64, 107, 116, 124).

Prioritizing collaboration with Indigenous leaders, strengthening intergenerational relationships and ensuring community involvement in program development and delivery are essential components for the success of HPV vaccination and cancer screening initiatives in high-income countries (97, 114). Community and peer support has been shown to positively contribute to overcoming negative attitudes toward cervical screening and vaccination cervical cancer screening barriers in Australia (120, 125), the United States (126) and Peru (127).

Finally, implementing mobile medical clinics to offer essential services has demonstrated its cost-effectiveness in overcoming access barriers for rural populations in low- and middle-income countries like South Africa (128) and Peru (129).

### 4.3 HPV self-testing implementation

Polymerase chain reaction (PCR)-based testing for high-risk HPVs on cervical or vaginal samples is now the gold standard for cervical cancer prevention (15). This screening method has shown higher sensitivity and negative predictive value compared to the Pap cytology test, thus allowing for larger intervals between screenings and providing greater protection against developing invasive cervical cancers (130–133). Another advantage of HPV testing is that self-collected vaginal samples can be used, which can reduce cost, is noninvasive, and thus emerges as a promising solution to increase screening participation in settings with limited resources, access to health services, or where cultural barriers exist (134–139). In particular, self-collected HPV testing has been shown to be better accepted among Indigenous women in Australia (108, 140), New Zealand (141, 142) and Guatemala (143). Accordingly, in recent years, the Ministry of Health of Guatemala has worked to implement self-collection for HPV testing as an alternative screening method to increase screening coverage in vulnerable populations, especially rural and Indigenous women (144, 145). To the best of available information, this is the only governmental initiative dedicated to improve cervical cancer prevention and control among Indigenous populations that has been implemented in a LAC country.

## 5 Conclusion

Because they experience higher levels of poverty and health inequities, Indigenous women worldwide are more vulnerable to cervical cancer than non-Indigenous women. In high-income countries, the disproportionately high burden of cervical cancer among Indigenous populations has been extensively characterized. Studies have consistently revealed a higher prevalence of HPV infection and increased incidence and mortality of cervical cancer among Indigenous women compared to their non-Indigenous counterparts. In these countries, targeted programs and initiatives aimed at overcoming the multiple and complex structural, social, and cultural barriers hindering Indigenous women’s access to cervical screening and HPV vaccination are yielding promising results.

On the contrary, in LAC, where Indigenous people constitute 10% of the population, the true extent of the burden of cervical cancer among Indigenous women remains largely unknown. Through an extensive review of published literature, this study presents the currently available data concerning the prevalence of cervical cancer and HPV infection among Indigenous women in LAC, highlighting the paucity of existing data and the critical gaps in research and healthcare interventions that need to be fill. While the findings discussed in this article offer valuable insights, one notable limitation is the absence of a meta-analysis, which could have facilitated a comparative assessment of HPV infection and cervical lesion prevalence rates between Indigenous women in LAC and the general population. Because indigenous status is not recorded in cancer registries of most LAC countries, accurately quantifying and understanding the burden of cervical cancer among Indigenous women in the region remains a challenge. Nevertheless, the limited available data suggest that Indigenous women in LAC face a heightened risk of cervical cancer incidence and mortality, as observed in Indigenous populations in high-income countries.

Strategies to improve cervical screening and HPV vaccination uptake for Indigenous women in LAC must be culturally sensitive, education-focused, and community-engaged. Detailed data on screening behaviors, vaccination uptake, and barriers specific to Indigenous populations in the region are essential. While taking example from successful policies and initiatives implemented in high-income countries, solutions adapted to the socio-economic realities of LAC countries and acknowledging the diversity among the more than 800 Indigenous groups in the region need to be found for bridging existing disparities and achieving the global goal of cervical cancer elimination.

## Data availability statement

The original contributions presented in the study are included in the article/supplementary material, further inquiries can be directed to the corresponding author.

## Author contributions

CM: Conceptualization, Funding acquisition, Writing – original draft, Writing – review & editing.
